# Study protocol for the Exercising Together© trial: a randomized, controlled trial of partnered exercise for couples coping with cancer

**DOI:** 10.1186/s13063-021-05548-3

**Published:** 2021-08-30

**Authors:** Kerri M. Winters-Stone, Karen S. Lyons, Nathan F. Dieckmann, Christopher S. Lee, Zahi Mitri, Tomasz M. Beer

**Affiliations:** 1 Knight Cancer Institute, School of Medicine, Oregon Health & Science University, 3181 SW Sam Jackson Park Road, Portland, OR 97239 USA; 2 School of Nursing, Oregon Health & Science University, Portland, OR USA; 3 Connell School of Nursing, Boston College, Chestnut Hill, MA USA; 4 Division of Psychology, School of Medicine, Oregon Health & Science University, Portland, OR USA

**Keywords:** Cancer survivor, Dyad, Caregiver, Exercise, Physical activity, Physical functioning, Quality of life, Inflammation, Cardiovascular health

## Abstract

**Background:**

Most cancer survivors are married, and cancer strains the physical and mental health of each partner and their intimate relationship. We created a partnered strength training program, Exercising Together©, where the survivor and his/her partner exercise as a team in order to improve physical and mental health of both members of the couple as well as the quality of their relationship. We have not yet determined if Exercising Together© is similarly effective in couples coping with different types of cancer nor if training as a team has unique and added benefits over those derived from supervised group training and/or shared behavior change. The purpose of this study is to determine the unique benefits of Exercising Together© on physical, mental, and relational health in couples coping with breast, prostate, or colorectal cancer.

**Methods:**

Survivors of prostate, breast and colorectal cancer (*N* = 294, 98 per cancer site) and their intimate, co-residing partners are recruited to participate in a single-blind, parallel group, randomized trial comparing three exercise groups that train twice per week for 6 months. Couples are randomized to one of three groups: (1) Exercising Together© where partners train as a team in a supervised group setting; (2) separate supervised group exercise classes for survivors or partners, respectively; (3) unsupervised home exercise program provided to each partner. The primary outcome is relationship quality (dyadic coping by the Dyadic Coping scale, emotional intimacy by the Dyadic Adjustment Scale, physical intimacy by the Physical Intimacy Behavior Scale, and symptom incongruence). Secondary outcomes are physical health (% body fat by DXA, serum fasting lipids (triglycerides, HDL, and LDL cholesterol), insulin resistance (HOMA-IR), resting blood pressure, C-reactive protein, TNF alpha, and physical functioning by the short Physical Performance Battery and SF-36) and mental health (depressive symptoms, anxiety, fear of recurrence) of each partner. Outcomes are collected at baseline, mid (3 months), post-intervention (6 months), and follow-up (12 months).

**Discussion:**

Exercising Together© could shift the paradigm of survivorship care toward novel couple-based approaches that could optimize outcomes for each partner because their health is interdependent on each other and their relationship.

**Trial registration:**

ClinicalTrials.govNCT03630354. Registered August 14, 2018

**Supplementary Information:**

The online version contains supplementary material available at 10.1186/s13063-021-05548-3.

## Background

Survivors of prostate (PC), breast (BC), and colorectal (CRC) cancer have a higher non-cancer mortality rate [1], greater incidence of cardiovascular disease [2, 3], and higher likelihood of functional limitations [4–8] compared to the general population. Most cancer survivors are married when diagnosed, so cancer also negatively impacts the health of the spouse and their relationship [1, 9, 10]. Spouses provide most of the supportive care to an ill partner and develop higher rates of hypertension, CVD, obesity, and mortality than spouses who are not caregivers [9–11]. High levels of psychological distress from the cancer experience [12–20] compound the impact of cancer on couple health where both cancer survivors and spouses experience higher rates of depressive symptoms, anxiety, and negative mood than age-matched controls [21, 22].

Since married, co-residing couples typically share environments, behaviors, and values; the health of one person can closely influence that of the other [20, 23, 24]. Spouses have a strong influence on each other’s health, which in turn impacts the quality of their relationship [20, 23, 24]. Cancer and the treatment thereof strains the marriage by hampering communication [25, 26] and interfering with sex [27, 28] which in turn erodes the emotional and physical intimacy that protects couples from the consequences of illness [29–36]. Communication avoidance and lack of intimacy can lead to high levels of incongruence about the illness (degree to which the survivor and spouse differ in their perceptions of the survivor’s function and symptoms) [37, 38] further challenging the couple’s ability to manage the illness together [39–42]. Thus, a major challenge for couples coping with cancer is to work together to maintain their physical and mental health, manage the illness, and restore balance to the relationship.

Current clinical approaches to support cancer survivors usually fall into two categories, individual physical rehabilitation or psychosocial interventions, neither of which is enough to improve the physical and mental health of both partners and their relationship all at once. In the marital literature, couple-based interventions are more effective than individual-based interventions for improving outcomes of both people [28, 43, 44], but these approaches do not address physical health [41]. Couples are unlikely to afford the time, effort and cost of multiple programs to tackle the threats cancer places on their individual health and marriage. But exercise could improve physical and mental health of both survivors and spouses [45, 46] and could also have relationship benefits if couples train as a team.

We have innovated an exercise strategy where the couple trains as a team to create a singular approach to simultaneously improve physical, mental, and relationship health of the couple. By fostering the skills couples need to exercise as a team, training can become a shared activity that promotes the support and communication that strengthens a relationship**.** Exercising Together© is a partnered strength training program designed to improve health and promote teamwork by the couple. Our pilot study of Exercising Together© performed in a group of PC couples (*n* = 64) was highly feasible (100% retention) and improved physical fitness, mood, and affectionate behavior [47–49]. We believe these outcomes occurred because we fostered skills within the couple to better collaborate, communicate, and support one another during exercise and that leads to better physical, mental, and relational health within the dyad. As a next step toward broader dissemination, we need to know if a couples-based exercise approach is similarly effective for couples coping with other cancers (and when survivor gender varies), early in the illness trajectory when a couple’s relationship is most vulnerable, and at reducing risk factors for chronic illness in both partners. An equally important next step is to distinguish the unique benefits of partnered training on individual and couple health from the possible benefits of exercising in a group with others and/or when partners both engage in a new health behavior.

The purpose of this study is to conduct a larger, more rigorous trial of Exercising Together© to other exercise delivery approaches that can separate the effects of teamwork from the effects of supervised group training and the effects of shared behavior change. The primary aim of the study is to determine the efficacy of Exercising Together© on relationship quality (intimacy, communication and symptom incongruence) in couples coping with PC, BC, or CRC. Secondary aims are to determine the efficacy of Exercising Together© on the physical health and mental health of both the survivor and spouse. We will also examine how long individual and couple-level benefits from *Exercising Together©* last and identify the types of couples that benefit most from partnered training*.* We hypothesize that Exercising Together© will significantly improve physical, mental, and relationship health of couples more than supervised, unpartnered exercise with other survivors or spouses, and unsupervised, unpartnered exercise and that these benefits will persist long-term.

## Methods

### Study design and setting

The Exercising Together© trial is a 3-arm, single-blind, parallel group, randomized trial where couples are allocated in a 1:1:1 ratio to the experimental arm of Exercising Together©, partnered exercise in a supervised group setting, or to one of two comparator arms: (1) supervised exercise where survivors and partners perform unpartnered exercise routines in a group with other survivors or partners, respectively, or (2) unsupervised, unpartnered exercise where both partners receive a training program to follow at home. All groups are expected to train twice weekly for 6 months and then are encouraged to continue training on their own for another 6 months. Data collection occurs at baseline, 3 months (midpoint of exercise), 6 months (end of supervised training), and at 1 year (6 months post-supervised training). Interventions and outcomes assessments will occur at Oregon Health & Science University (OHSU) in Portland, Oregon. Exercise classes may also be held at community sites throughout Oregon and southwest Washington to help improve accessibility to the program and enhance recruitment of a broader and more diverse group of participants. The study is approved by the OHSU IRB (#18000) and is registered with ClinicalTrials.gov (NCT03630354). Any modification of this protocol must be documented in the form of a protocol revision or amendment signed by the principal investigator and approved by the OHSU Knight Cancer Institute and the IRB before the revision or amendment may be implemented. The only circumstance in which the amendment may be initiated without regulatory approval is for a change necessary to eliminate an apparent and immediate hazard to the patient. In that event, the investigator must notify the IRB in writing within 5 working days after the implementation.

### Sample

Participants are PC, BC, or CRC survivors and their co-residing spouse or partner. Eligible survivors must meet the following inclusion criteria: (1) have received a diagnosis of PC, BC, or CRC without evidence of metastatic disease; (2) be 3 years or less from their diagnosis date; and (3) completed primary treatment (surgery, radiation, and/or chemotherapy) at least 6 weeks prior to enrollment. Concurrent adjuvant hormone therapy is permitted and must have been initiated ≥ 6 weeks prior to enrollment. Both survivors and partners must meet the following criteria: (1) be aged 35–80 years old, (2) co-residing with each other in an intimate relationship, and (3) not regularly engaging in 2 or more strength training sessions (30 min per session at a moderate-vigorous intensity) per week over the previous month. We exclude couples where one or both partners has any of the following: (1) cognitive difficulties that preclude answering the survey questions, participating in the exercise classes or performance tests, or providing informed consent; (2) a medical condition, movement or neurological disorder, or medication use that contraindicates participation in moderate intensity exercise; (3) inability to attend > 75% of the intervention classes due to conflict with the designated time of day, days of the week, and/or location for the exercise class; or (4) incapable of answering survey questions, participating in class, following directions during performance testing, and providing informed consent when English is the language used. All survivors must receive medical clearance for participation in moderate intensity exercise. Partners must also receive medical clearance if indicated by responses to the American College of Sports Medicine pre-participation screening questions [50] or at the discretion of the Principal Investigator.

### Power and sample size

Required sample size was derived for the outcomes of aim 1. Power analysis using traditional assumptions of the repeated measures ANOVA model suggest that as few as 174 couples would be necessary to detect a small effect (*d* = .20) between groups over time, an effect smaller than the observed effect on affectionate behavior in spouses in our previous work [47]. However, these traditional power calculations are known to be optimistic. While there are good power formulae for MLM clustered designs and repeated measures designs, as yet there is no specific formula for calculating power for dyadic analyses. Using a formula provided by Raudenbush and [51, 52] for individual repeated measures and estimates from our previous dyadic models, we calculate a sample of 264 couples measured 4 times over a 12-month period has a power of 0.80 to detect a moderate effect (*d* = .40) on change over time. As dyad models using MLM control for interdependence between members of the couple, power is increased over an individual model. No universal approach has been adopted for sample size considerations in GMM and this will be used in an exploratory aim; however, our *n*-to-items ratio exceeds sample size recommendations for related approaches (10–20:1). To insulate the sample size against an estimated attrition of 10% across the study period, 294 couples will be randomized. The attrition estimate is more conservative than our prior trial of *Exercising Together* where no couples in the exercise program dropped out. We will recruit approximately even numbers of couples coping with each type of cancer (*N* = 98 per disease site).

### Recruitment and retention

We have planned for a 36-month enrollment period to recruit 294 couples into the proposed study (~ 8 couples/month). Couples enroll into 1 of 10 waves (a new wave begins every 3–4 months) of ~ 30 couples per wave to maintain reasonable class sizes and make efficient use of testing resources. Our primary recruitment strategy is through mailings to potential participants identified through the Oregon State Cancer Registry (OSCaR), a successful approach used in previous studies. In addition to OSCaR, we also send letters to patients identified uniquely through the OHSU hospital cancer registry, e-mail messages delivered through MyChart to OHSU patients and by clinician referral. Community-based recruitment occurs via print ads, radio, social media, and presentations at cancer organizations and conferences.

Up to 20% of participants in exercise oncology trials drop out within 12 weeks, although the rates are lower when an intervention is provided to every group [53]. We expect strong recruitment and retention in this study since exercise is provided to every participant (i.e., no waitlist or usual care control group), close free parking is provided, and exercise classes are conducted at community locations to be more convenient for where participants live.

### Procedures

**Fig. 1:**
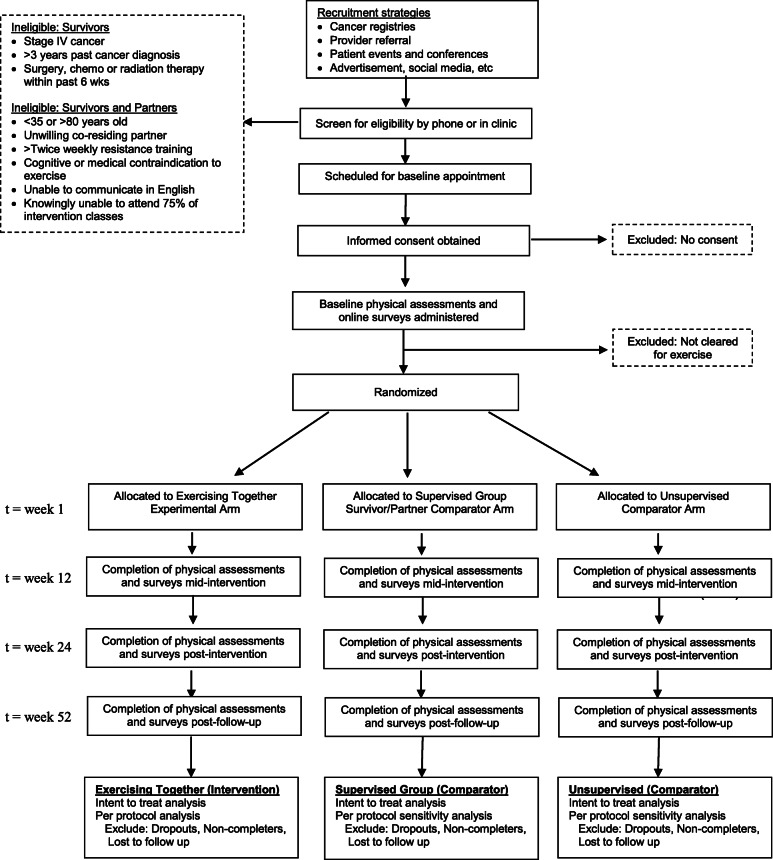
Planned participant flow through the trial

The planned flow of participants in the study is outlined in Fig. 1. Couples who express interest in the study are screened for eligibility either by phone or in person by study staff. After initial screening, potentially eligible couples are scheduled to go to OHSU for consent and a baseline visit consisting of body composition assessment, physical performance testing, venous blood sampling, and initial survey completion. Couples complete written surveys on a computer at baseline and online for follow-up visits, unless they prefer to complete paper surveys. Staff review surveys for completeness and follow-up with participants in person or by phone on missing data. The same measurements taken at baseline are repeated at 3-, 6-, and 12-month follow-up visits. Unless otherwise stated, measures are completed by both members of the couple at each timepoint. All study outcome assessors are blinded to group assignment, which occurs after baseline testing. If a couple drops out of the research, their data will be retained for analysis but no more data will be collected past the point of withdrawal. The investigator may choose to withdraw a participant without their consent if their health changes and the study is no longer in their best interest, if new information becomes available, if she/he does not follow the study rules, and/or if the study is stopped by the IRB. If one partner of a couple is unable or unwilling to continue exercise training, any data collected on them prior to their withdrawal will be used for purposes of the study. Data will be continued to be collected on the other partner and they will be allowed to continue in the exercise program. If needed, their program will be adapted so that it can be performed independently.

### Randomization and blinding

Participants will be randomly assigned to 1 of 3 groups in a 1:1:1 allocation ratio: (1) Exercising Together©, (2) separate unpartnered group exercise, and (3) separate unpartnered home exercise. To avoid confounding that could occur due to differential exercise tolerance based on age, randomization will be stratified by average couple age (< 60 vs. 60–80 years). The biostatistician (ND) uses a computer-generated (MS Excel) random numbers table to allocate participant ID numbers to study arms in blocks of 6–9 couples per study wave to ensure even assignment across waves. Individual assignments are placed into sealed envelopes prior to enrollment and assigned in the order that couples are scheduled for testing. After completion of baseline testing couples are provided with the sealed envelope that contains their randomly assigned group.

### Study interventions

Regardless of group assignment, all participants are expected to engage in two 1-h exercise sessions per week for 6 months. For supervised exercise training, class size is limited to 9–10 couples assigned to Exercising Together© or 9–10 survivors or partners assigned to separate group classes. To maintain a trainer to participant ratio of ~ 1:10 so that enough individual attention is given to participants to ensure proper form and safety, Exercising Together© will be taught by two instructors who will then each teach a survivor or partner group class. The additional instructor in Exercising Together© is also needed to ensure delivery and supervision of the teamwork component of that intervention (described below). Couples assigned to unsupervised, unpartnered training at home will have two face-to-face training sessions with a trainer to learn the study exercise program and then be provided a written manual and video to follow at home. To ensure safety of participants and quality control over intervention delivery within and across instructors, every trainer must have a fitness certification and complete 2-day training workshop that covers how to instruct each exercise protocol, training progression, safety considerations, and research conduct specific to the exercise program. Additionally, each trainer will follow a written training protocol and will be regularly observed by study staff who monitor participant retention, exercise compliance, and instruction fidelity.

The basic training program for all three study arms is a functional resistance training program, based upon our prior studies in cancer survivors [48, 54–56], that target physical functioning and that use exercises which could be performed unpartnered or partnered and with free weights or resistance bands to aim for equality across study arms (Tables 1 and 2). Across all study arms, our functional resistance training program is comprised of a fundamental set of compound movements that involve more than one joint emphasizing muscle groups used in every day activities, plus functional exercises that directly mimic every day movements. A greater emphasis on increasing muscle strength is incorporated into supervised classes because more equipment can be used under supervision. Supervised classes use a combination of free weights (weight vest and dumbbells) and resistance bands, whereas the unsupervised program uses a combination of resistance bands and body weight to ensure safety when exercising alone. Dumbbells are used for upper body exercises (e.g., row, chest press) and the deadlift and weight is prescribed to reach target repetition maximum (RM), where the weight is set by the maximum repetitions properly performed at a given weight (i.e., 12RM). Weighted vests are used for lower body resistance exercises (e.g., squats, step ups) and prescribed as a percent of body weight added to the vest in order to reach a target RM. Since functional training is more kinesthetically challenging for novice exercisers and depends upon proper form and safety, over the first 4 weeks of the supervised programs we incorporate exercises to develop postural alignment to promote stabilization in joints and muscles used in core exercises [57]. Fundamental exercises are performed at a moderate-vigorous intensity, progressing across a range of moderate intensity and increasing volume over the first 3 months, then toward more vigorous intensity over the last half of training and/or as tolerated. Training volume (intensity, sets, reps) is gradually progressed over time in order to maintain continuous overload toward a final goal of 2–3 sets per exercise at 8–10 RM (Table 1). The initial training progression across the study is based upon our prior work and pilot study of Exercising Together*©* [49, 58, 59] (Table 2). In addition to fundamental strengthening exercises, 2–3 functional movements, including core muscle exercises, are incorporated into every class within a study program (e.g., lunges, planks, bridges). The home-based training program includes both fundamental and functional exercises used in the supervised training programs performed with exercise bands of varying resistance to provide progressive overload.

**Table 1:** Training progression for fundamental strengthening exercises in supervised study arms

	Intensity	Reps	Sets
Month 1	12–15RM	12–15	1–2
Month 2	12–15RM	12–15	2
Month 3	12–15RM	12–15	2–3
Month 4	10–12RM	10–12	2–3
Month 5	8–12RM	8–12	2–3
Month 6	8–10RM	8–10	2–3

**Table 2:** Partnered and unpartnered versions of functional exercises in supervised programs

Target region	Partnered version	Unpartnered version
Total body	Slam ball w/ pass to partner*	Slam ball to ground/wall*
Total body/core	Partnered plank with clap	Plank w/ shoulder tap
Core	Partnered hand press	Stationary twist*
Core	Partnered leg throw down	Dead bug
Core/lower body	Bridge w/ partner resistance	Bridge*
Lower body	Reverse lunge w/ partner foot tap	Reverse lunge with foot tap
Lower body	Synchronized band side step*	Band side step*
Upper + lower body	Partner wall sit + partnered row*	Wall sit w/ band pull*

*Resistance applied by exercise bands, blocks, or slam balls. Resistance bands increased every 1–2 months as tolerated

### Exercising Together© training

**Fig. 2:**
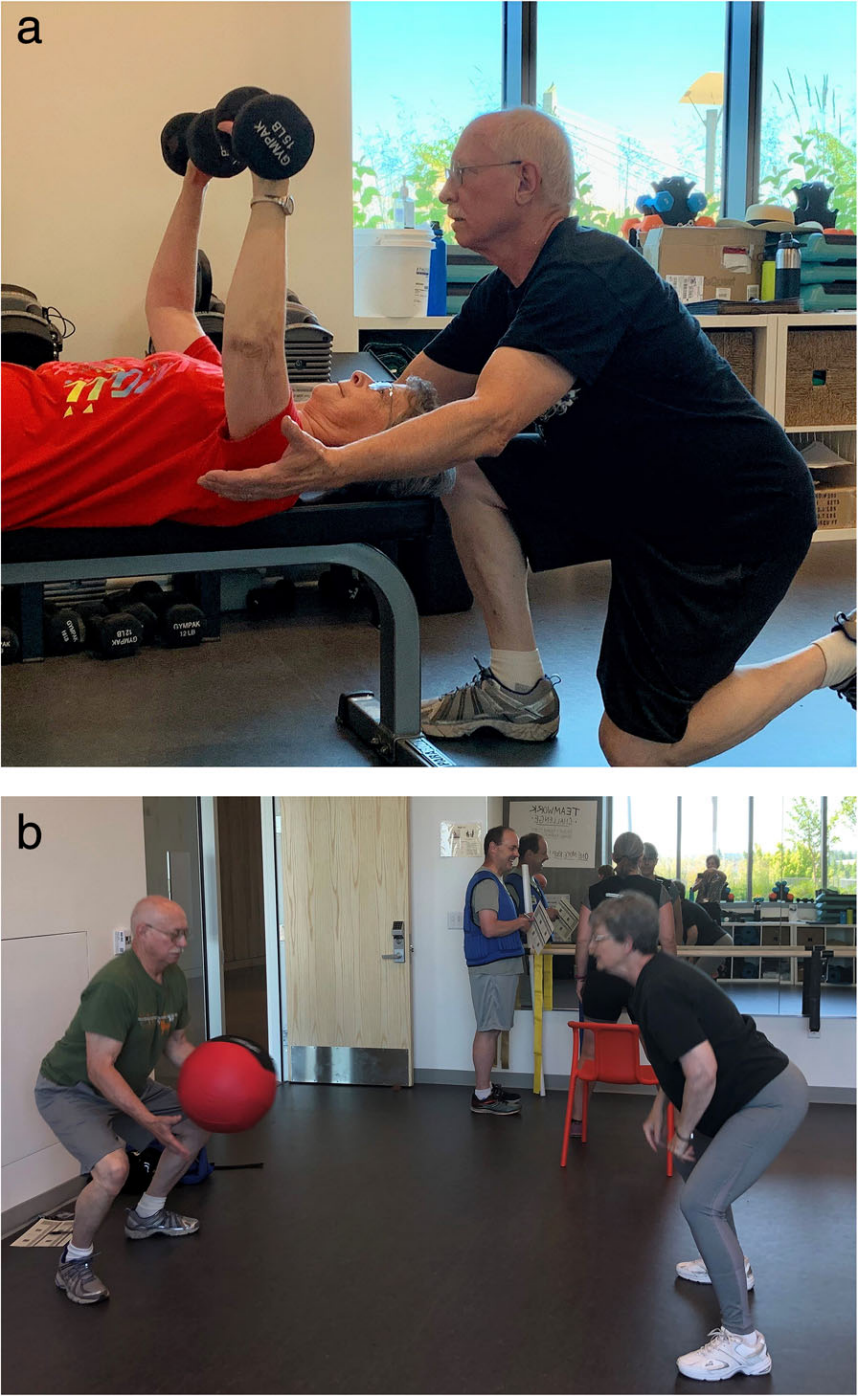
**a** A prostate cancer survivor coaching his spouse during a chest press exercise. **b** The same couple performing a functional exercise together

The Exercising Together© intervention is conducted in a supervised group setting and consists of a partnered strength training program designed to promote relationship, physical, and mental health by having couples train as a team. We hypothesize that the teamwork skills used when training will permeate outside of the exercise setting and enhance the overall relationship of the couple. We foster teamwork (communication, motivation, support, trust) a couple uses as they collaborate toward a common goal (e.g., improve their health and functioning) by having couples practice coaching each other, collaborate on tandem exercises, and incorporate teamwork principles during training. During fundamental strengthening exercises, couples practice teamwork by having one partner assume the role of “coach” while the other exercises and then partners switch roles (Table 3, Fig. 2a). During fundamental exercises, coaches, and partners are encouraged to practice teamwork skills within each role (Table 3). We also have couples collaborate together by having participants perform 1–2 tandem exercises per session where the couple must work together to complete the exercise (Fig. 2b) and require the couple to communicate and interact both non-verbally and verbally with one another. The first 12 weeks of the program focuses on mastering form and initial progression of the fundamental exercises and building teamwork through coaching roles and functional exercises. During the second half of the program, we increase the emphasis on teamwork by specifically teaching principles of effective teams and having couples incorporate them into exercise sessions (Table 3). Couples are encouraged to incorporate the teamwork skills they use during exercise to their day to day interactions outside of training.

**Table 3:** Methods for building teamwork in Exercising Together© through practicing skills as coach and exerciser and incorporating principles into daily behavior

Teamwork practice	
Skill (role)	Examples
Assess (Coach)	• Determine partner’s ability to do the session exercises • Adjust intensity and reps based on session goals
Assist (Coach)	• Help partner with position and use of proper technique/form • Count repetitions
Applaud (Coach)	• Verbal encouragement during and after exercise
Advise (Coach)	• Discuss how exercise session went (e.g., too hard, too easy) • Determine goal for next session
Receive (Exerciser)	• Listen to instruction, feedback, and praise from coach
Respond (Exerciser)	• Disclose any concerns, limitations (e.g., fatigue) before and during exercise • Change performance in response to feedback • Discuss accomplishments and goals for next session
Teamwork principles	
**Principle**	**Example**
Communication	Openly receive a critique/correction from your coach and respond positively. Be “coachable”.
Support/commitment	Transition from the stress of the day to focus on being fully present for your partner.
Motivation/encouragement	Use a non-verbal way to celebrate or congratulate each other for training accomplishments, e.g., a high five and fist bump.
Trust/respect	When giving a critique/correction during coaching, also give a compliment/reward

### Supervised group training (unpartnered)

Couples randomized to this arm attend separate supervised, group exercise classes for survivors only and for partners only. Survivor and partner classes run at the same time and location to prevent time-of-day confounding. Each group performs the same set of fundamental and functional exercises as that described in Exercising Together©, but unpartnered and without any element of teamwork (Table 2). Survivor and partner classes are each taught by a single trainer.

### Unsupervised training

Couples randomized to this study arm are taught a home-based version of the supervised, unpartnered program to do at home on their own using body weight and resistance bands. Within 2 weeks of randomization, each couple has two 1-h training sessions with an exercise trainer who teaches the functional strength training program, modified to their abilities and safety in an unsupervised setting. We provide participants with an instructional video (secure web channel for streaming or DVD version) of the training program to follow at home. The trainer checks in weekly by phone during the first month, and once per month thereafter to collect attendance, and to assess tolerance, promote progression of resistance bands, and modify programs as needed. Participants can perform the program on days and at times convenient for them as long as they allow 48 h between sessions. There is no requirement that partners exercise at the same time, though we track if couples exercise concurrently to potentially account for this in analyses.

### Participant safety and side effects

Any form of exercise carries a slight risk of injury. We will take steps to reduce the risk of injury and other issues that might limit compliance, including (1) use of certified fitness trainers to instruct exercise programs, (2) requirement of physician clearance for every cancer survivor and for partners when indicated, and (3) monitoring and early care of musculoskeletal symptoms which may include slight adjustments in the training program (modifying intensity or select exercises) with a goal to maintain the overall training stimulus. If a survivor develops metastatic disease during the study his/her data from the point of the diagnosis will be considered separately, but she/he could continue in the study program with physician clearance.

### Six-month follow-up period

To evaluate the persistent effects of Exercising Together© on individual and relationship health, all couples will be followed for an additional 6 months after formal training stops. To provide couples in supervised programs a resource to continue to engage in exercise after supervised training stops we will provide them with video, accessible through a secure web channel for streaming or DVD, of their programs to use at home. During the last month of supervised training, we will prepare participants for the transition to unsupervised training with discussions about behavioral strategies to stay active. Couples in the unsupervised program will be encouraged to continue exercising on their own at home. We will repeat all measures at month 12 in order to better assess the residual effects of Exercising Together© among couples that do or do not continue to exercise or that engage in shared activities or not in the follow-up period.

### Measures

#### Primary outcome

The focus of this study is the impact of Exercising Together© on relationship quality, assessed in the following ways:

1. *Dyadic coping*. The dyadic coping measure consists of two subscales (active engagement and protective buffering )[60, 61].. Active engagement assesses the extent to which the survivor and spouse view their partner’s active involvement and support [60, 61]. Participants respond to five items using a Likert scale from 1 (never) to 5 (very often). Higher scores indicate higher levels of perceived active engagement. Protective buffering assesses the extent to which the survivor and spouse view their partner’s use of hiding concerns and denying worries [60, 61]. Participants respond to six items using a Likert scale from 1 (never) to 5 (very often). Higher scores indicate higher levels of perceived protective buffering. The scale has exhibited high Cronbach’s alpha values (.75 to .87) in studies of couples with cancer [62].
2. *Emotional intimacy*. The Dyadic Adjustment Scale [63] is measure of emotional intimacy with 32-items that use a 6-point Likert scale. Higher scores indicate better dyadic adjustment. The scale has demonstrated good internal consistency, *α* = .90–.94 [64], including in couples with cancer [18, 65, 66]. Sensitivity, specificity, and validity of the scale have been supported [63].
3. *Physical intimacy*. The Physical Intimacy Behavior scale has a person self-report, on a 1–4 scale, the frequency that they engage in, initiate, and avoid four affectionate (i.e., touching, kissing, hugging, caressing) and two sexual (i.e., sexual intercourse, foreplay) behaviors. Subscales have demonstrated strong internal consistency and construct validity [67].
4. *Concealment of symptoms*. Patient concealment of symptoms (i.e., hiding feelings to prevent the partner from experiencing distress about the illness) will be measured using the Emotional-intimacy Disruptive Behavior Scale [67]. Participants report the extent to which they engage in 8 behaviors using a scale from 1 (rarely or none of the time) to 4 (most or all of the time). High scores indicate greater concealment. Items on the scale have strong agreement (*α* = .83) and the instrument has good evidence for construct validity [67].
5. *Symptom incongruence*. Survivor-spouse incongruence regarding three common treatment-related symptoms will be assessed by having survivors and partners rate survivor symptoms at each time point using the following instruments:
  - *Pain* using the Brief Pain Inventory (BPI). The BPI consists of 2 subscales, pain intensity and pain interference [68]. Values from items within each subscale are averaged together to yield scores 0–10. Low values indicate no pain and high values represent worst possible pain.
  - *Fatigue* in the past 7 days using the Functional Assessment in Chronic Illness Therapy (FACIT) fatigue questionnaire [69]. Summed scores of the 13 items range from 0 to 52 with higher scores indicating less fatigue.
  - *Physical function* using the physical function subscale of the SF-36 medical outcomes survey. Scores range from 0 to 100 where low scores indicate difficulty performing daily activities independently and 100 indicating no difficulty in performing daily activities [70].

#### Secondary outcomes: objective measures

##### Body composition

Total bone-free lean and fat mass (kg) is assessed by dual energy x-ray absorptiometry (DXA) (Hologic-QDR Discovery Wi; APEX software, v.4.02) performed by trained research staff. The coefficient of variation for body composition measures in our lab is < 1.0% [71]. We will also quantify visceral and subcutaneous fat mass (kg) from the whole body scan. Visceral adipose tissue measured by DXA is highly correlated (*r* = 0.93) and linearly related to visceral adipose tissue from CT scans [72], but DXA exposes patients to considerably less radiation and is less time and cost intensive.

##### Cardiovascular health

Fasting blood lipids (total, high-density and low-density lipoprotein; cholesterol; triglycerides) and insulin resistance (Homeostasis Model of Assessment: HOMA) will be measured as markers of cardiovascular health. All markers will be processed and quantified by the OHSU Core Laboratory using commercially available kits. We will also measure resting blood pressure (SunTech CT40) in accordance with the 2005 report on blood pressure determination [73].

##### Inflammation

Fasting serum levels of high sensitivity C-reactive protein (hsCRP) and tumor necrosis factor alpha (TNF alpha) will be analyzed by the OHSU Core Laboratory using a standard ELISA kit.

##### Objective physical functioning

The Physical Performance Battery (PPB) consists of 3 timed tests: time to complete 5 repeated chair stands (s), ability to keep balance during increasingly difficult stances, and usual walk speed over 4 meters (m/s). Scores on each test are converted to a 0–4 scale and then summed, so that total PPB scores range from 0 to 12. Low scores on the PPB are associated with subsequent mobility disability, inability to complete activities of daily living, hospitalization, nursing home admission, and mortality [74–77]. The PPB is reliable, sensitive to change, and has established normative values [78].

#### Secondary outcomes: patient-reported measures

##### Quality of life (general population)

The SF-36 will be used to measure health-related quality of life in both survivors and partners. The SF-36 has 8 subscales: perceived physical function, role limitations due to physical problems, social functioning, bodily pain, general mental health, role limitations due to emotional problems, vitality, and general health perceptions. Scores range from 0 to 100 for overall quality of life and on the subscales, where higher scores indicate better functioning. The SF-36 has good evidence for validity in cancer, in addition to other chronic conditions [79].

##### Quality of life (cancer)

The QLQ-C30 is the preferred quality of life measure for cancer patients in clinical trials [80] and is also administered to survivors in this study. The QLQ-C30 includes both function and symptom scales and an overall quality of life measure. Scores range from 0 to 100 for overall quality of life and on the subscales, where higher scores indicate better functioning. The instrument has strong evidence for reliability and validity both for individual subscales and the summary score [80, 81].

##### Depressive symptoms

The Center for Epidemiological Studies-Depression (CES-D) scale will be used to measure the degree of depressive symptoms [82]. Scores range from 0 to 60, with higher scores indicating greater number of symptoms that occur more often. The CES-D scale has been widely used, demonstrating sensitivity, specificity, validity [82–84], and internal consistency [20, 85–87].

##### Anxiety

The PROMIS anxiety short form 8a measure will be used to measure anxiety over the past 7 days, using a 5-pt. Likert scale ranging from 1 (never) to 5 (always). Higher scores represent higher levels of anxiety. The items included have strong evidence for validity and are sensitive to change [88].

##### Fear of recurrence

The Fear of Recurrence scale [89] measures the amount of concern survivors have about their cancer returning in the future. A modified version of the scale has been developed for family members. The measure has strong internal consistency in survivors [90] and family members [91].

##### Caregiver strain

Completed by spouse/partners only, the 18-item Multidimensional Caregiver Strain Index measures subjective perceptions of stress related to the caregiving role [92]. Scores range from 18 to 90 with higher scores indicating greater (worse) strain.

#### Descriptive variables and additional measures of interest

##### Demographic variables, cancer history (survivor), health habits, and anthropometric data

Demographic variables, cancer history (survivor), health habits, and anthropometric data (height, weight) will be obtained at all visits. Presence of chronic medical conditions, used to characterize the health of the sample, will be measured by the Charlson Comorbidity Index [93], a weighted index originally developed to predict mortality. Shared activities at each time point will be measured using two items developed and used by this team. Survivors and partners are asked to rate engagement in (a) leisure activities together and (b) exercise activities together on a 0–4 scale, with a follow-up open-ended question about the types of activities that partners completed together.

##### Fidelity of exercise training

Evidence that the functional resistance training programs in each study arm effectively increased muscle strength will be evaluated by 1-repetition maximum testing for leg press and chest press. The testing will be conducted according to established protocols [94]. The coefficient of variation for this measure within our laboratory is 0.05–0.06 [71].

##### Adherence

Adherence to the exercise intervention, as measured by the percentage of prescribed sessions completed, will be tracked from attendance logs completed by the exercise instructor in supervised classes and will be collected on monthly check-ins for couples assigned to unsupervised training. Adherence data will be used to describe the dose of exercise received by participants. Make-up sessions using a written plan or video tape will not be counted in adherence estimates.

##### Exercise outside the exercise intervention

Exercise outside the exercise intervention could affect the secondary outcomes in our study. Participation in moderate-vigorous intensity physical activity (kcal/week) will be measured by the 41-item Community Healthy Activities Model Program for Seniors (CHAMPS) physical activity questionnaire [57]. CHAMPS is a frequently used, highly reliable [58] measure of physical activity in older adults, including studies in cancer survivors performed by our team [29, 42, 59, 60]. We will also look at individual items to see whether participants significantly increase participation in other types of exercise in addition to their assigned study program.

##### Adverse events

To capture adverse events, a survey is administered monthly during a participants’ yearlong participation in the study. If an adverse event reported through this survey indicates that their reporting condition is due to a study-related exercise activity or if more information is needed to determine reportability, study staff will follow-up with a phone call or e-mail. Participants will also have the opportunity to report adverse events during exercise class or at physical performance measurement appointments. Adverse events will be graded according to their significance for severe consequences, such as injury or death, using the following grades: mild, moderate, or severe and classified as unrelated, possibly related or related to the study exercise programs.

### Data safety and monitoring

The OHSU Knight Cancer Institute Data and Safety Monitoring Committee (DSMC) is responsible for overall coordination of all aspects of the data safety and monitoring plan filed with the OHSU IRB. The internal audit team conducts quality assurance audits on all open clinical trials that are not monitored by another source. The initial audit is conducted once enrollment commences and yearly thereafter. An interim safety review occurs early in the intervention period, after the first 45 enrolled couples (~ 1/8th of total sample) have completed 3 months of exercise training, to assess early for program safety. The DSMC meets once each month to review the audit team’s progress and findings and to review significant adverse events and/or unanticipated problem reports, and Interim Analysis reports. The DSMC also reviews a full report of study activity for all local, active clinical trials at the time of continuing review submission including protocol amendments, revisions, and consent form revisions; interim analysis results; protocol violations; total number of patients enrolled on-study as compared to expected numbers; and all unanticipated problems submitted (including dates, description, and relationship). The DSMC oversees the process of serious adverse event reporting to assure that reporting requirements are met.

### Data management and analysis plan

Standard institutional practices will be followed as described in the OHSU Information Security and Research Data Resource Guide (http://ozone.ohsu.edu/cc/sec/isg/res_sec.pdf) to maintain the confidentiality and security of data collected in this study. A copy of the consent form and documentation of consent will be maintained in the participant’s medical record as well as stored in a study file kept in a locked cabinet (for paper documents only) or stored on an encrypted and password protected computer drive in the OHSU Knight Cancer Research Building (KCRB). All protected health information collected from the study either directly from participants or via their electronic health record will be stored on an encrypted and password protected computer drive in the OHSU KCRB that only IRB approved persons have access to. All other data collected for this study will be stored in OHSU installation of REDCap, a highly secure and robust web-based research data collection and management system. Any surveys that were filled out on paper will be stored in a locked cabinet in a locked, secure room in the OHSU KCRB. Data will be stored until data analysis is complete and then the data will be transferred to a repository.

In order to ensure data quality, all data are entered into an electronic system that includes either discrete range limits and/or requires double data entry. Descriptive statistics and graphs will be used to check for any departures from statistical assumptions (e.g., normality, outliers) [95]. We will examine dropout and patterns of missing data to determine mechanisms (missing completely at random (MCAR), missing at random (MAR), or not ignorable). In the case of data missing MCAR or MAR, model-based maximum likelihood estimation available in the multi-level modeling (MLM) approach will allow unbiased parameter estimation using all available data (i.e., missing data is handled efficiently with no loss of information) [96, 97]. If the rate of attrition is high and missing data is not ignorable, we will continue with the planned analyses but temper our conclusions. We will conduct intention-to-treat (ITT) and completers only analyses. ITT analyses will include all participants regardless of whether or not they complete all assessments and/or of adherence to exercise. Based on median attendance data from our prior trials, completers will be defined as participating in 50% or more of the scheduled exercise sessions. We will track medical treatment changes, cancer recurrence, and adherence to exercise training. Age, comorbidities, and time since diagnosis will be considered as covariates in all analyses. All analyses will be conducted in R, Hierarchical Linear Modeling (HLM), and MPlus v7.2 statistical software packages.

#### Primary outcome analysis

A longitudinal multivariate-outcomes dyad model will be used to directly examine couple trajectories in intimacy (emotional and physical) and communication over time. This is a multi-level model where responses from the survivor and partner are modeled simultaneously to control for the interdependent nature of the data and autocorrelation among repeated assessments. The within-dyad model has four coefficients representing intercepts (baseline assessments) and slopes (rates of change) for survivors and partners that become outcome variables in a between-dyad model. Models with linear and quadratic change across time will be compared to determine best fit to the data. Change in the primary outcome will be addressed with between-dyad models that include dummy variables to directly examine the effects of the partnered intervention vs. supervised individual (dummy 1) and unsupervised individual (dummy 2) exercise groups on individual level changes (controlling for couple effects) in intimacy and communication across time. A significant group coefficient on the slopes in the between-dyad model and significantly better fitting model (evidenced by deviance statistic) will indicate the rate of change across time is different depending on a treatment group (i.e., interaction effect). We will also explore the efficacy of *Exercising Together©* on symptom incongruence (regarding survivor pain and fatigue) in couples. Univariate-outcomes models will be used to generate Empirical Bayes estimates of the gap between survivor and partner over time for each symptom measure. This comprehensive approach to estimating incongruence has been described elsewhere [98–100]. The effect of *Exercising Together©* on incongruence will be directly tested (similar to above model) by a significant coefficient for one of the two group variables on the slope parameter and significantly better fitting model.

#### Secondary outcome analysis

Separate longitudinal multivariate-outcomes models will be used to directly examine the effect of partnered strength training in couples on each physical (body composition, lipids, insulin resistance, blood pressure, inflammation, and physical function) and mental (anxiety, depressive symptoms, fear of recurrence) health outcome as described previously. Sustained effects will be tested by comparing linear and quadratic trajectories across time (as discussed above). We will also separately estimate and directly compare the intervention effects between the intervention (baseline to 6 months) and follow-up (6 months to 12 months).

#### Analysis to determine patterns and predictors of types of couples who benefit the most

Latent growth mixture modeling (LGMM) identifies distinct patterns of change that vary around different means and have unique variance and homogenous within-trajectory growth [101]. Based on conditional probabilities, cases are assigned to the “most likely class” or pattern of change over time (e.g., couples who benefit/improve most from the intervention). Couple-level estimates from analyses above will be integrated into progressive LGMM to determine if there are distinct and naturally occurring patterns of change in outcomes over time with the dyad as the unit of analysis. Survivor-, partner-, and couple-level determinants of fitting one pattern of change over the other(s) will be modeled using logistic, multinomial, or ordinal regression as appropriate. This integrated multi-level and mixture modeling approach has been used previously by this team [102], as it allows us to identify types of dyads and differentiate them based on individual and couple-level factors.

## Discussion

PC, BC, and CRC cancer survivors account for 51% of the 16.9 million cancer survivors in the USA [103] — a figure expected to grow by 10 million in less than a decade. Nearly all of these cancer survivors are aging adults and are expected to live many years, if not decades, after their cancer diagnosis, raising concerns about the impact of cancer on their long-term health, work ability, and health care expenses. Most cancer survivors are married/partnered when diagnosed and cancer will also threaten the physical and mental health of their aging spouse and the quality of their marital relationship. Thus, the societal and economic impact of cancer goes far beyond an individual experience. Spouse caregivers experience significant health declines, such as increased CVD risk [104, 105], and are at greater risk for mobility limitations [106] and mortality than other family caregivers [9–11]. Since husbands and wives typically share environments, behaviors, and values the health and wellbeing of couples becomes closely intertwined, where the physical and mental health of one partner influences the others’ [20, 23, 24] as well as their satisfaction with the relationship [107]. Poor relationship quality alone increases the risk for CVD and mortality [108–111], building the imperative to find novel ways to foster the supportive nature of the couple with cancer. There is no singular program yet in practice that addresses the triple threat of declines in survivor and spouse health (physical and mental) and in their marital relationship.

Exercising Together© has the couple train as a team with the expectation that the teamwork skills that partners use to exercise together permeate outside of the training room. Our pilot study of this 6-month partnered training program in 64 couples had no dropout at all and improved physical and mental health outcomes and showed signs of improving the relationship. Since our pilot, only two other small studies in couples coping with cancer have been published but neither tested a partnered exercise approach [112, 113]. Both studies reported better improvements in survivor mood when the spouse also exercised but did not include any objective measures of physical health in survivors nor any outcomes for partners. Couple outcomes were barely assessed but in one study where couples exercised together, i.e., dancing, the survivor reported increased levels of dyadic trust, whereas in another study, if couples only engaged in the same exercise program performed separately, partner support remained unchanged. Collectively, these studies point to increasing benefits as the couple is more engaged in collaborative exercise, with Exercising Together© showing the most promise for improving partner, spouse, and relationship health altogether.

Exercising Together© is an innovative, partnered exercise-based approach unlike any other because it simultaneously targets the physical and mental health of the survivor, his/her spouse, and their relationship**.** Several features of our intervention and design contribute to the innovation of this trial. First, our partnered exercise approach maximizes the benefits of exercise on individual and relationship health by fostering the skills couples need to work as a team and collaborate toward a common goal by communicating, motivating, and supporting each other during training. We anticipate that couples will use these skills outside of the exercise environment, furthering the impact of partnered training on couple health. Second, our study design includes individual exercise comparison arms to isolate the unique effect of teamwork in Exercising Together from the potential effects of exercising in a group (i.e., social effect of being with other survivors or spouses) or of exercise itself. Third, this larger trial includes cancers where the gender of survivor and spouse vary allowing us to examine the influence of gender and role on study outcomes. Finally, the use of innovative modeling approaches will strengthen our ability to determine how much couples benefit from Exercising Together© and which couples benefit the most.

If successful, the Exercising Together© trial will have a high impact on the field of cancer survivorship since the number of aging married cancer survivors will double over the next 20 years**.** Clinical practice is bereft of evidence-based programs that simultaneously target the physical and mental health of the survivor, his/her spouse, *and* their relationship. Exercising Together© could shift the paradigm of survivorship care toward novel couple-based approaches that could optimize outcomes for each partner because their health is interdependent on each other and their relationship. This innovative program has the potential to broaden beyond cancer to other illnesses that could greatly increase the impact of this work.

### Trial status

At the date of publication the current protocol version is 1.0. Recruitment for the trial began in January 2019 and is expected to complete by March 2022.

## Supplementary Information

**Additional file 1.** Consent and Authorization Form - Survivor

**Additional file 2.** Consent and Authorization - Partner

## Data Availability

The datasets used during the current study are available from the corresponding author on reasonable request.

## Abbreviations

ACSM: American College of Sports Medicine
BC: Breast cancer
CHAMPS: Community Healthy Activities Model Program for Seniors
CRC: Colorectal cancer
DSMC: Data and Safety Monitoring Committee
DXA: Dual energy X-ray absorptiometry
EORTC QLQ-C30: European Organization for Research and Treatment of Cancer Quality of Life Questionnaire
FACIT: Functional Assessment of Chronic Illness Therapy
GMM: Growth mixture modeling
ITT: Intent to treat
KCRB: Knight Cancer Research Building
MET: Metabolic equivalent
OHSU: Oregon Health & Science University
OSCaR: Oregon State Cancer Registry
PC: Prostate cancer
PPB: Physical Performance Battery
REDCap: Research Electronic Data Capture

## References

[1] Baade PD, Fritschi L, Eakin EG (2006). Non-cancer mortality among people diagnosed with cancer (Australia). Cancer Causes Control..

[2] Patnaik JL, Byers T, DiGuiseppi C, Denberg TD, Dabelea D (2011). The influence of comorbidities on overall survival among older women diagnosed with breast cancer. J Natl Cancer Inst.

[3] Bardia A, Arieas E, Zhang Z, DeFilippis A, Tarpinian K, Jeter S (2012). Comparison of breast cancer recurrence risk and cardiovascular disease incidence risk among postmenopausal women with breast cancer. Breast Cancer Res Treat..

[4] Sweeney C, Schmitz KH, Lazovich D, Virnig BA, Wallace RB, Folsom AR (2006). Functional limitations in elderly female cancer survivors. J Natl Cancer Inst..

[5] Hewitt MRJ, Yancik R (2003). Cancer survivors in the United States: age, health, and disability. Gerontol A Biol Sci Med Sci..

[6] Kurtz ME, Kurtz JC, Stommel M, Given CW, Given B (2001). Physical functioning and depression among older persons with cancer. Cancer Pract..

[7] Given B, Given C, Azzouz F, Stommel M (2001). Physical functioning of elderly cancer patients prior to diagnosis and following initial treatment. Nurs Res..

[8] Keating NL, Norredam M, Landrum MB, Huskamp HA, Meara E (2005). Physical and mental health status of older long-term cancer survivors. J Am Geriatr Soc..

[9] Vitaliano PP, Zhang J, Scanlan JM (2003). Is caregiving hazardous to one's physical health? A meta-analysis. Psychol Bull..

[10] Fredman L, Bertrand RM, Martire LM, Hochberg M, Harris EL (2006). Leisure-time exercise and overall physical activity in older women caregivers and non-caregivers from the Caregiver-SOF Study. Prev Med..

[11] Li Q, Loke AY (2013). A spectrum of hidden morbidities among spousal caregivers for patients with cancer, and differences between the genders: a review of the literature. Eur J Oncol Nurs..

[12] Hagedoorn M, Sanderman R, Coyne JC, Bolks HN, Tuinstra J (2008). Distress in couples coping with cancer: a meta-analysis and critical review of role and gender effects. Psychol Bull..

[13] Lewis FM, Fletcher KA, Cochrane BB, Fann JR (2008). Predictors of depressed mood in spouses of women with breast cancer. J Clin Oncol..

[14] Northouse LL, Templin T, Mood D, Oberst M (1998). Couples' adjustment to breast cancer and benign breast disease: a longitudinal analysis. Psycho-oncology..

[15] Shands ME, Lewis FM, Sinsheimer J, Cochrane BB (2006). Core concerns of couples living with early stage breast cancer. Psycho-oncology..

[16] Tuinstra J, Hagedoorn M, Van Sonderen E, Ranchor AV, Van den Bos GAM, Nijboer C (2004). Psychological distress in couples dealing with colorectal cancer: gender and role differences and intracouple correspondence. Br J Health Psychol..

[17] Badr H, Carmack CL, Kashy DA, Cristofanilli M, Revenson TA (2010). Dyadic coping in metastatic breast cancer. Health Psychol..

[18] Milbury K, Badr H, Carmack CL (2012). The role of blame in the psychosocial adjustment of couples coping with lung cancer. Ann Behav Med..

[19] Zwahlen D, Hagenbuch N, Jenewein J, Carley MI, Buchi S (2011). Adopting a family approach to theory and practice: measuring distress in cancer patient-partner dyads with the distress thermometer. Psycho-oncology..

[20] Lyons KS, Bennett JA, Nail LM, Fromme EK, Dieckmann N, Sayer AG (2014). The role of patient pain and physical function on depressive symptoms in couples with lung cancer: a longitudinal dyadic analysis. Journal of Family Psychology..

[21] Costanzo ES, Ryff CD, Singer BH (2009). Psychosocial adjustment among cancer survivors: findings from a national survey of health and well-being. Health Psychol..

[22] Mitchell AJ, Ferguson DW, Gill J, Paul J, Symonds P (2013). Depression and anxiety in long-term cancer survivors compared with spouses and healthy controls: a systematic review and meta-analysis. Lancet Oncol..

[23] Bourassa KJ, Memel M, Wollverton C, Sbarra DA (2015). A dyadic approach to health, cognition, and quality of life in aging adults. Psychol Aging..

[24] Hagedoorn M, Buunk BP, Kuijer RG, Wobbes T, Sanderman R (2000). Couples dealing with cancer: role and gender differences regarding psychological distress and quality of life. Psycho-oncology..

[25] Manne S, Ostroff J, Winkel G, Grana G, Fox K (2005). Partner unsupportive responses, avoidant coping, and distress among women with early stage breast cancer: patient and partner perspectives. Health Psychol..

[26] Manne S, Badr H, Zaider T, Nelson C, Kissane D (2010). Cancer-related communication, relationship intimacy, and psychological distress among couples coping with localized prostate cancer. J Cancer Surviv..

[27] Sanders S, Pedro LW, Bantum EO, Galbraith ME (2006). Couples surviving prostate cancer: long-term intimacy needs and concerns following treatment. Clin J Oncol Nurs..

[28] Reese JB, Keefe FJ, Somers TJ, Abernethy AP (2010). Coping with sexual concerns after cancer: the use of flexible coping. Supportive Care Cancer..

[29] Galinsky AM, Waite LJ (2014). Sexual activity and psychological health as mediators of the relationship between physical health and marital quality. J Gerontol B Psychol Sci Soc Sci.

[30] Gilbert E, Ussher JM, Perz J (2010). Renegotiating sexuality and intimacy in the context of cancer: the experiences of carers. Arch Sex Behav.

[31] Beck AM, Robinson JW, Carlson LE (2009). Sexual intimacy in heterosexual couples after prostate cancer treatment: what we know and what we still need to learn. Urol Oncol..

[32] Rolland JS (1994). In sickness and in health: The impact of illness on couples' relationships. J Marital Fam Ther..

[33] Birditt K, Antonucci TC (2008). Life sustaining irritations? Relationship quality and mortality in the context of chronic illness. Soc Sci Med..

[34] Umberson D, Williams K, Powers DA, Liu H, Needham B (2006). You make me sick: marital quality and health over the life course. J Health Soc Behav..

[35] Coyne JC, Smith DA (1991). Couples coping with a myocardial infarction: a contextual perspective on wives' distress. J Pers Soc Psychol..

[36] Zakowski SG, Harris C, Krueger N, Laubmeier KK, Garrett S, Flanigan R, Johnson P (2003). Social barriers to emotional expression and their relations to distress in male and female cancer patients. Br J Health Psychol..

[37] Miller LM, Lyons KS, Bennett JA (2015). Incongruent perceptions of pain and physical function among famillies living with lung cancer. Supportive Care Cancer..

[38] Lyons KS, Jones KD, Bennett RM, Hiatt SO, Sayer AG (2013). Couple perceptions of fibromyalgia symptoms: the role of communication. PAIN®.

[39] Zhang AY, Siminoff LA (2003). The role of the family in treatment decision making by patients with cancer. Oncol Nurs Forum..

[40] Broberger E, Tishelman C, von Essen L (2005). Discrepancies and similarities in how patients with lung cancer and their professional and family caregivers assess symptom occurrence and symptom distress. J Pain Symptom Manage..

[41] Berg CA, Upchurch R (2007). A developmental-contextual model of couples coping with chronic illness across the adult life span. Psychol Bull..

[42] McCarthy MJ, Lyons KS (2015). Incongruence between stroke survivor and spouse perceptions of survivor functioning and effects on spouse mental health: a mixed-methods pilot study. Aging Ment Health..

[43] Martire LM, Lustig AP, Schulz R, Miller G, Helgeson VS (2004). Is it beneficial to involve a family member? A meta-analysis of psychosocial interventions for chronic illness. Health Psychol.

[44] Northouse LL, Williams AL, Given BA, McCorkle R (2012). Psychosocial care for family caregivers of patients with cancer. J Clin Oncol..

[45] Friedenreich CM, Orenstein MR (2002). Physical activity and cancer prevention: etiologic evidence and biological mechanisms. J Nutr..

[46] Centers for Disease Control. Physical activity and good nutrition: essential elements to prevent chronic diseases and obesity 2003. Nutr Clin Care. 2003;6(3):135-8. https://pubmed.ncbi.nlm.nih.gov/14979458/. 14979458

[47] Lyons KS, Winters-Stone KM, Beer TM (2016). The effects of partnered exercise on physical intimacy in couples coping with prostate cancer. Health Psychol..

[48] Winters-Stone KM, Lyons KS, Nail LM, Beer TM (2012). The Exercising Together project: design and recruitment for a randomized, controlled trial to determine the benefits of partnered strength training for couples coping with prostate cancer. Contemp Clin Trials..

[49] Winters-Stone KMLK, Dobek J, Nail L, Bennett JA (2016). Beer TM Benefits of partnered strength training for prostate cancer survivors and spouses: results from a randomized controlled trial of the Exercising Together project. J Cancer Surviv..

[50] Riebe D, Franklin B, Thompson P, Garber C, Whitfield G, Magal M (2015). Updating ACSM’s recommendations for exercise preparticipation health screening. Med Sci Sports Exerc..

[51] Raudenbush SWBA (2002). Hierarchical linear models.

[52] Raudenbush SW, X-F L (2001). Effects of study duration, frequency of observation, and sample size on power in studies of group differences in polynomial change. Psychol Methods.

[53] Steins Bisschop CN, Courneya KS, Velthuis MJ, Monninkhof EM, Jones LW, Friedenreich C, van der Wall E, Peeters PHM, May AM (2015). Control group design, contamination and drop-out in exercise oncology trials: a systematic review. PLoS One..

[54] Winters-Stone KM, Dobek J, Bennett JA, Nail LM, Leo MC, Schwartz A (2012). The effect of resistance training on muscle strength and physical function in older, postmenopausal breast cancer survivors: a randomized controlled trial. J Cancer Surviv..

[55] Winters-Stone KM, Dobek JC, Bennett JA, Dieckmann NF, Maddalozzo GF, Ryan CW, Beer TM (2015). Resistance training reduces disability in prostate cancer survivors on androgen deprivation therapy: evidence from a randomized controlled trial. Arch Phys Med Rehabil..

[56] Winters-Stone KM, Li F, Horak F, Luoh SW, Bennett JA, Nail L (2012). Comparison of tai chi vs. strength training for fall prevention among female cancer survivors: study protocol for the GET FIT trial. BMC Cancer.

[57] Cook G, KK LB, Rose G, Bryant MF (2010). Introduction to Screening and Assessment. Movement functional movement systems: screening, assessment and corrective strategies.

[58] Winters-Stone K, Dobek J, Nail L, Bennett JA, Naik A, Schwartz A (2011). Strength training stops bone loss and builds muscle in postmenopausal breast cancer survivors: a randomized controlled trial. Breast Cancer Res Treat..

[59] Winters-Stone KM, Dobek JC, Bennett JA, Maddalozzo GF, Ryan CW, Beer TM (2014). Skeletal response to resistance and impact training in prostate cancer survivors. Med Sci Sports Exerc..

[60] Hagedoorn M, Kuijer RG, Wobbes T, Sanderman R (2000). Marital satisfaction in patients with cancer: does support from intimate partners benefit those who need it the most?. Health Psychol.

[61] Buunk BP, Berkhuysen MA, Sanderman R, Nieuwland W, Ranchor AV (1996). Actieve betrokkenheid, beschermend bufferen en overbescherming: Meetinstrumenten voor de role van de partner bij hartrevalidatie. [The role of the partner in heart disease: Active engagement, protective buffering, and overprotection]. Gedrag & Gezondheid..

[62] Hinnen C, Hagedoorn M, Sanderman R, Ranchor AV (2007). The role of distress, neuroticism and time since diagnosis in explaining support behaviors in partners of women with breast cancer: Results of a longitudinal analysis. Psycho-oncology..

[63] Spanier GB (1976). Measuring dyadic adjustment: New scales for assessing the quality of marriage and similar dyads. J Marriage Fam..

[64] Badr HJ (2004). Coping in marital dyads: a contextual perspective on the role of gender and health. Personal Relationships..

[65] Carmack Taylor CL, Badr H, Lee JH, Fossella F, Pisters K, Gritz ER, Schover L (2008). Lung cancer patients and their spouses: psychological and relationship functioning within 1 month of treatment initiation. Ann Behav Med..

[66] Morgan MA, Small BJ, Donovan KA, Overcash J, McMillan SC (2011). Cancer patients with pain: the spouse/partner relationship and quality of life. Cancer Nurs..

[67] Druley JA, Stephens MAP, Coyne JC (1997). Emotional and physical intimacy in coping with lupus: women's dilemmas of disclosure and approach. Health Psychol..

[68] Cleeland CS, Ryan KM (1994). Pain assessment: global use of the Brief Pain Inventory. Ann Acad Med Singapore..

[69] Yellen SB, Cella D, Webster K, Blendowski C, Kaplan E (1997). Measuring fatigue and other anemia-related symptoms with the Functional Assessment of Cancer Therapy (FACT) measurement system. J Pain Symptom Manage..

[70] Ware JE, Sherbourne CD (1992). The MOS 36-Item Short-Form Health Survey (SF-36). I. Conceptual framework and item selection. Med Care..

[71] Winters KM, Snow CM (2000). Detraining reverses positive effects of exercise on the musculoskeletal system in premenopausal women. J Bone Miner Res..

[72] Micklesfield LK, Goedecke JH, Punyanitya M, Wilson KE, Kelly TL (2012). Dual-energy X-ray performs as well as clinical computed tomography for the measurement of visceral fat. Obesity (Silver Spring)..

[73] Pickering TG, Hall JE, Appel LJ, Falkner BE, Graves J, Hill MN, Jones DW, Kurtz T, Sheps SG, Roccella EJ (2005). Recommendations for blood pressure measurement in humans and experimental animals: part 1: blood pressure measurement in humans: a statement for professionals from the Subcommittee of Professional and Public Education of the American Heart Association Council on High Blood Pressure Research. Circulation..

[74] Ibrahim EM, Al-Homaidh A (2011). Physical activity and survival after breast cancer diagnosis: meta-analysis of published studies. Med Oncol..

[75] Kenfield SA, Stampfer MJ, Giovannucci E, Chan JM (2011). Physical activity and survival after prostate cancer diagnosis in the health professionals follow-up study. J Clin Oncol..

[76] Meyerhardt JA, Giovannucci EL, Holmes MD, Chan AT, Chan JA, Colditz GA, Fuchs CS (2006). Physical activity and survival after colorectal cancer diagnosis. J Clin Oncol..

[77] Richman EL, Kenfield SA, Stampfer MJ, Paciorek A, Carroll PR, Chan JM (2011). Physical activity after diagnosis and risk of prostate cancer progression: data from the cancer of the prostate strategic urologic research endeavor. Cancer Res..

[78] Guralnik J, Simonsick E, Ferrucci L, Glynn R, Berkman L, Blazer D (1994). A short physical performance battery assessing lower extremity function: association with self-reported disability and prediction of mortality and nursing home admission. J Gerontol..

[79] Ware JE, Sherbourne CD (1992). The MOS 36-item short-form health survey (SF-36):I. Conceptual framework and item selection. Med Care..

[80] Aaronson NK, Ahmedzai S, Bergman B, Bullinger M, Cull A, Duez NJ, Filiberti A, Flechtner H, Fleishman SB, JCJM H, Kaasa S, Klee M, Osoba D, Razavi D, Rofe PB, Schraub S, Sneeuw K, Sullivan M, Takeda F (1993). The European Organization for Research and Treatment of Cancer QLQ-C30: a quality-of-life instrument for use in International Clinical Trials in Oncology. J Natl Cancer Inst..

[81] Giesinger JM, Kieffer JM, Fayers PM, Groenvold M, Petersen MA, Scott NW, Sprangers MA, Velikova G, Aaronson NK, EORTC Quality of Life Group (2016). Replication and validation of higher order models demonstrated that a summary score for the EORTC QLQ-C30 is robust. J Clin Epidemiol..

[82] Radloff LS (1977). The CES-D Scale: A self-report depression scale for research in the general population. Appl Psychol Meas..

[83] Beekman ATF, Deeg DJH, Van Limbeek J, Braam AW, De Vries MZ, Van Tilburg W (1997). Criterion validity of the Center for Epidemiological Studies Depression Scale (CES-D): results from a community-based sample of older subjects in the Netherlands. Psychol Med..

[84] Radloff LS, Teri L (1986). Use of the Center for Epidemiological Studies-Depression Scale with older adults. Clin Gerontol..

[85] Lyons KS, Stewart BJ, Archbold PG, Carter JH, Perrin N (2004). Pessimism and optimism as early-warning signs for compromised health in Parkinson's disease caregiving. Nurs Res..

[86] Robbins ML, Lopez AM, Weihs KL, Mehl MR (2014). Cancer conversations in context: naturalistic observation of couples coping with breast cancer. J Fam Psychol..

[87] Siminoff LA, Wilson-Genderson M, Baker S (2010). Depressive symptoms in lung cancer patients and their family caregivers and the influence of family environment. Psycho-oncology..

[88] Pilkonis PA, Choi SW, Reise SP, Stover AM, Riley WT, Cella D, PROMIS Cooperative Group (2011). Item banks for measuring emotional distress from the Patient-Reported Outcomes Measurement Information System (PROMIS(R)): depression, anxiety, and anger. Assessment..

[89] Northouse L (1981). Mastectomy patients and the fear of cancer recurrence. Cancer Nurs..

[90] Hilton BA (1989). The relationship of uncertainty, control, commitment, and threat of recurrence to copin strategies used by women diagnosed with breast cancer. J Behav Med..

[91] Mellon S, Northouse LL (2001). Family survivorship and quality of life following a cancer diagnosis. Res Nurs Health..

[92] Stull D (1996). The Multidimensional Caregiver Strain Index (MCSI): its measurement and structure. J Clin Geropsychology..

[93] Charlson ME, Pompei P, Ales KL, MacKenzie CR (1987). A new method of classifying prognostic comorbidity in longitudinal studies: development and validation. J Chronic Dis..

[94] Franklin B, Medicine ACoS (2009). ACSM's guildelines for exercise testing and prescription.

[95] Tukey JW (1977). Exploratory data analysis.

[96] Little R, Rubin D (1987). Statistical analysis with missing data.

[97] Schafer J, Graham JW (2002). Missing data: our view of the state of the art. Psychol Methods..

[98] Raudenbush SW, Brennan RT, Barnett RC (1995). A multivariate hierarchical model for studying psychological change within married couples. J Fam Psychol..

[99] Sayer AG, Klute MM, Bengtson VL, Acock AC, Allen KR, Dilworth-Anderson P, Klein DM (2005). Analyzing couples and families: Multilevel methods. Sourcebook on Family Theory and Research.

[100] Cano A, Johansen AB, Franz A (2005). Multilevel analysis of couple congruence on pain, interference, and disability. PAIN®.

[101] Lee CS, Faulkner KM, Thompson JH (2020). Identifying subgroups: Part 2: Trajectories of change over time. Eur J Cardiovasc Nurs.

[102] Lee CSL, K.S. (2019). Patterns, relevance, and predictors of dyadic mental health over time in lung cancer. Psycho-Oncology..

[103] American CS (2018). Cancer Facts & Figures 2018.

[104] Ji J, Zöller B, Sundquist K, Sundquist J (2012). Increased risks of coronary heart disease and stroke among spousal caregivers of cancer patients. Circulation..

[105] Kim Y, Carver CS, Shaffer KM, Gansler T, Cannady RS (2015). Cancer caregiving predicts physical impairments: roles of earlier caregiving stress and being a spousal caregiver. Cancer..

[106] Fredman L, Cauley JA, Satterfield S, Simonsick E, Spencer SM, Ayonayon HN, Harris TB, Health ABC Study Group (2008). Caregiving, mortality, and mobility decline: the health, aging, and body composition (health abc) study. Arch Intern Med..

[107] Zhou ES, Kim Y, Rasheed M, Benedict C, Bustillo NE, Soloway M, Kava BR, Penedo FJ (2011). Marital satisfaction of advanced prostate cancer survivors and their spousal caregivers: the dyadic effects of physical and mental health. Psycho-Oncology..

[108] Hooker SA, Grigsby ME, Riegel B, Bekelman DB (2015). The impact of relationship quality on health-related outcomes in heart failure patients and informal family caregivers: an integrative review. J Cardiovasc Nurs.

[109] Liu H, Waite L (2014). Bad marriage, broken heart? Age and gender differences in the link between marital quality and cardiovascular risks among older adults. J Health Soc Behav..

[110] Rohrbaugh MJ, Shoham V, Coyne JC (2006). Effect of marital quality on eight-year survival of patients with heart failure. Am J Cardiol..

[111] Uchino BN, Bosch JA, Smith TW, Carlisle M, Birmingham W, Bowen KS, Light KC, Heaney J, O'Hartaigh B (2013). Relationships and cardiovascular risk: perceived spousal ambivalence in specific relationship contexts and its link to inflammation. Health Psychol..

[112] Demark-Wahnefried W, Jones LW, Snyder DC, Sloane RJ, Kimmick GG, Hughes DC, Badr HJ, Miller PE, Burke LE, Lipkus IM (2014). Daughters and Mothers Against Breast Cancer (DAMES): main outcomes of a randomized controlled trial of weight loss in overweight mothers with breast cancer and their overweight daughters. Cancer..

[113] Kamen C, Heckler C, Janelsins MC, Peppone LJ, McMahon JM, Morrow GR, et al. A Dyadic exercise intervention to reduce psychological distress among lesbian, gay, and heterosexual cancer survivors. LGBT Health. 2016;3(1):57–64. 10.1089/lgbt.2015.0101 PMC4770846 26652029

