# Supplementary material for: Study protocol for the Exercising Together© trial: a randomized, controlled trial of partnered exercise for couples coping with cancer
Source: Trials. 2021 Aug 30;22:579. doi: 10.1186/s13063-021-05548-3 (PMC8404361; doi:10.1186/s13063-021-05548-3)
Supplement: Supplementary file 1 — Additional file 1. Consent and Authorization Form - Survivor [file 13063_2021_5548_MOESM1_ESM.docx]

**OHSU Knight Cancer Institute Consent and Authorization Form: Survivor**

#### Title: EXERCISING TOGETHER^©^ for Couples Coping with Cancer

**Funded by:** National Institutes of Health (NIH)

**Conflict of Interest Statement:** There are no conflicts of interests to disclose

#### Table of Contents

[Introduction 2](#_Toc530560454)

[Purpose 2](#_Toc530560455)

[Procedures 3](#_Toc530560456)

[Risks 5](#_Toc530560457)

[Benefits 6](#_Toc530560458)

[Privacy 6](#_Toc530560459)

[Participation 8](#_Toc530560460)

[Signature
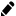
 11](#_Toc530560461)

**Study Contact Information**

| **Purpose** | **Role** | **Contact Name** | **Contact Phone Number** | **Email** |
| --- | --- | --- | --- | --- |
| For medical questions about the study | Principal Investigator | Kerri Winters-Stone, PhD | 503-494-0813 | wintersk@ohsu.edu |
|  | Co-Investigator | Tom Beer, MD | 503-494-4394 | beert@ohsu.edu |
| For non-medical questions about the study | Study Coordinator | Carolyn Guidarelli | 503-346-0307 | borsch@ohsu.edu |
| For questions about research in general | Ethics Committee | ORIO | 503-494-7887 | [irb@ohsu.edu](mailto:irb@ohsu.edu) |
| For 24-hour medical emergencies | 911 | Emergency Dispatch | 911 |  |
|  | Oncologist On-Call | OHSU Operator | 503-494-8311 |  |

#

# Introduction

**What is the usual approach to my** **CANCER?**

You are being asked to take part in this study because you are 35-80 years old, have had treatment for either prostate, breast or colorectal cancer and currently reside with a spouse/partner.

Treatments for cancer can cause side effects such as fatigue as well as strain on their relationship. People who do not take part in this study may receive recommendations, such as encouragement to exercise, and/or ways to adjust their daily activities to help combat some of the physical and emotional side effects of cancer treatment. ​

**What are my other choices if I do not take part in this study?**

If you decide not to take part in this study, you have other choices. For example:

- you may choose to take part in a different study, if one is available

# Purpose

**Why is this study being done?**

The purpose of this study is to determine the effect of a partnered resistance training program on relationship quality as well as physical and emotional health in prostate, breast and colorectal cancer survivors and their spouse/partner.

We are asking you to provide information for a data bank, also called a repository. This information will be stored indefinitely and may be used and disclosed in the future for research.

This is a clinical trial, a type of research study. Medical personnel who carry out research studies are called “investigators.” The investigator will explain the clinical trial to you. Clinical trials include only people who choose to take part. Please take your time to make your decision about taking part. You can discuss your decision with your friends and family. You can also discuss it with your health care team or another doctor. If you have any questions, ask the investigator.

**How many people will take part in this study?**

As many as 294 couples will take part in this study which will be conducted at Oregon Health & Science University (OHSU).

# Procedures

**What are the study groups?**

You will be randomized into one of three study groups.

1. Couples exercise (both partners exercise together in a group setting)
2. Separate survivor and spouse group exercise (each partner exercises in a group with either other survivors OR other spouses)
3. Separate survivor and spouse home exercise (each partner receives an individual home exercise program)

“Randomization” means you will be put into a group by chance. Neither you nor the investigator can choose the group you will be in. You have an equal chance of being placed in any of the three groups.

**How long will I be in this study?**

You will be in the study for about 12 months.

**What tests and procedures will I have if I take part in this study?**

Participants who are randomized will be asked to complete two 1-hour resistance exercise sessions per week, either at a facility or at home depending on group assignment, for the next six months. We will collect information about your cancer diagnosis and treatment from your medical records. If you have a prolonged absence during the study period you may not be able to continue the study exercise sessions if, in the opinion of the study team, it is not appropriate for you to continue in an ongoing, progressive exercise program

After your six months of participation in the study exercise sessions this portion of the study will be complete. We will ask you to complete a short survey at that time to provide feedback about your experience in the exercise program. During the next six months of the study, you can choose whether or not to continue to exercise on your own and will not be discouraged from doing so. We will ask you to report your exercise during this time via a survey at 9 and 12 months past your study enrollment.

All participants will undergo a testing session at the beginning of the study, and again at 3 months, 6 months, and 12 months. At each testing session, you will complete a set of questionnaires that are designed to assess your health status, physical activity habits and relationship with your spouse/partner. They should take approximately one hour to complete and you may refuse to answer any question you do not feel comfortable with. A short survey will also be sent monthly that will address any health events that may have occurred in the past 4 weeks. It will take about 10 minutes or less to complete.

During your testing sessions at OHSU we will also ask you to complete a series of physical tests described as follows:

1. Lift as much weight with your legs as you can in one try.
2. Lift as much weight with your arms as you can in one try.
3. Walk at your usual walking pace for about 8 feet.
4. Rise and sit from a chair five times as quickly as you can.
5. Try to maintain your balance while standing on the floor.

6. You will have 6 small movement sensors placed on your body using a light strap and asked to maintain your balance while standing on the floor and walk up to 6 minutes.

7. You will be given a DXA scan that will measure the muscle, fat, and bone content of your body. A DXA scan is a type of x-ray. You must lie still on a table for about 5 minutes during this procedure. Women who can become pregnant must have a negative pregnancy test before the DXA scan is performed.

8. You will be asked to give small amount of blood (~1 tablespoon) to measure markers of inflammation and cardiovascular health. The blood test requires you to fast, abstain from smoking cigarettes, drinking alcohol, and drinking caffeinated beverages for at least 12 hours before your appointment.

9. You will have your blood pressure taken three times while sitting quietly in a chair.

The physical tests together will take about 1-2 hours to complete. The entire testing session will take about 2-3 hours per couple.

This schedule lists the study procedures:

| **Procedure** | **M1** | **M2** | **M3** | **M4** | **M5** | **M6** | **M7** | **M8** | **M9** | **M10** | **M11** | **M12** |
| --- | --- | --- | --- | --- | --- | --- | --- | --- | --- | --- | --- | --- |
| DXA Scan | X |  | X |  |  | X |  |  |  |  |  | X |
| Blood Pressure | X |  | X |  |  | X |  |  |  |  |  | X |
| Blood Draw | x |  | x |  |  | x |  |  |  |  |  | x |
| Physical Tests | x |  | x |  |  | x |  |  |  |  |  | x |
| Questionnaires | X |  | X |  |  | X |  |  | X |  |  | X |
| Monthly Survey | X | X | X | X | X | X | X | X | X | X | X | X |
| Resistance Training Exercise | x | x | x | x | x | x |  |  |  |  |  |  |
| **Approx Total Time** | **10-11 hr** | **8 hr** | **10-11 hr** | **8 hr** | **8 hr** | **10-11 hr** | **10 min** | **10min** | **25 min** | **10 min** | **10 min** | **2-3 hr** |

# Risks

**What possible risks can I expect from taking part in this study?**

In this study you will be exposed to radiation during the DXA scans. While no radiation dose has been determined to be entirely safe, the amount to which you will be exposed is not known to cause health problems. Before each DXA scan, female participants who can become pregnant must have a urine pregnancy test, unless you have undergone surgical removal of your ovaries and/or uterus (hysterectomy) or are postmenopausal (complete absence of menstrual cycles for one year or longer). We are required to ensure that you are not pregnant before you receive the scan because of the exposure to x-rays. The reason we do this is to be as careful as possible to not scan a woman who is pregnant. Although the body DXA scan has a very low dose of radiation, it could affect a developing unborn child. The results of the pregnancy test will remain private. We will inform you of the results and, if positive, refer you to your regular doctor or health care provider for ongoing care but you will no longer be eligible to continue in this research study.

We will draw blood from a vein on your arm. You may feel some pain when your blood is drawn. There is a small chance the needle will cause bleeding, a bruise, or an infection.

There are some discomforts and risks from the physical tests. In order to try to minimize risks, all testing will be conducted by trained personnel. You may feel sore after the physical tests. Muscle soreness usually goes away after two or three days. You may sustain an injury during the physical testing. The risk of this is low and the researchers are trained to show you the best ways to avoid injury during the tests.

As part of this study, you will be asked to complete questionnaires. Some of these questions may seem very personal or embarrassing. They may upset you. You may refuse to answer any of the questions that you do not wish to answer. If the questions make you very upset, we will help you to find a counselor.

As with any form of regular exercise training, the risk of injury is increased. We have taken precautions to make the exercises as safe as possible. The exercises will be led by a certified exercise professional. The study exercises have been performed before in cancer survivors as well as women aged 30-85 years who have reported no significant injuries as a result of the study.

Here are important points about how you and the investigator can make these risks less of a problem:

- Tell the investigator if you are unable to complete a test or a survey. The investigator can allow for a longer rest period during the test or reschedule it for a different day
- Tell the investigator if you are unwilling to complete a test or survey. You may choose to opt-out of some or all tests and surveys.

Let your investigator know of any questions you have about possible side effects. You can ask the investigator questions about side effects at any time.

# Benefits

**What possible benefits can I expect from taking part in this study?**

Participants may not benefit from participating in this study; however, their participation may contribute to knowledge used for future studies that address patients with cancer and their families.

# Privacy

Access to your test results

We plan to share the results of the DXA scan with you at the end of the study.

Who will see my medical information?

We will take steps to keep your personal information confidential, but we cannot guarantee total privacy.

We will create and collect health information about you as described in the WHY IS THIS STUDY BEING DONE and the What tests and procedures will I have if I take part in this study sections of this form. Health information is private and is protected under federal law and Oregon law. By agreeing to be in this study, you are giving permission (also called authorization) for us to use and disclose your health information as described in this form.

The investigators, study staff and others at OHSU may use the information we collect and create about you in order to conduct and oversee this research study, store it in a repository, and conduct future research.

We may release this information to others outside of OHSU who are involved in conducting or overseeing this research, including:

- The Office of Human Research Protections (OHRP), a federal agency that oversees research in humans
- The National Cancer Institute (NCI)

Those listed above may also be permitted to review and copy your records, including your medical records.

We may also share your information with other researchers, who may use it for future research studies.

We will not release information about you to others not listed above, unless required or permitted by law. We will not use your name or your identity for publication or publicity purposes, unless we have your special permission.

To help us protect your privacy, we have obtained a Certificate of Confidentiality from the National Institutes of Health.  With this Certificate, the researchers can refuse to disclose information that may identify you, even by a court subpoena, in any federal, state, or local civil, criminal, administrative, legislative, or other proceedings.  The researchers will use the Certificate to resist any demands for information that would identify you, except as explained below.

The Certificate cannot be used to resist a demand for information from personnel of the United States Government that is used for auditing or evaluation of federally funded projects.

A Certificate of Confidentiality does not prevent you or a member of your family from voluntarily releasing information about yourself or your involvement in this research.  If an insurer, employer, or other person obtains your written consent to receive research information, then the researchers may not use the Certificate to withhold that information.

However, if we learn about abuse of a child or elderly person or that you intend to harm yourself or someone else, or about certain communicable diseases, we will report that to the proper authorities.

When we send specimens or information outside of OHSU, it may no longer be protected under federal or Oregon law. In this case, your information could be used and re-released without your permission.

After all of the analyses are completed using your collected data, some participant data, including your contact

information, will be stored in a private locked-repository managed by Dr. Kerri Winters-Stone, the Principal

Investigator of this study. A code number will be assigned to your data, as well as to information about you.

Only the investigators named on this consent form will be authorized to link the code number to you. Other

investigators who may receive your data for research will be given only the code number which will not

identify you.

We may continue to use and disclose your information as described above indefinitely.

# Participation

**Can I stop taking part in this study?**

Yes. You can decide to stop at any time. If you decide to stop for any reason, it is important to let the investigator know as soon as possible so you can stop safely. Another reason to tell your investigator that you are thinking about stopping is to discuss what testing, follow-up, or additional treatment could be most helpful for you. If you stop, you can decide whether or not to let the investigator continue to provide your medical information to the organization running the study.

The investigator will tell you about new information or changes in the study that may affect your health or your willingness to continue in the study.

The investigator may take you out of the study:

- If your health changes and the study is no longer in your best interest
- If new information becomes available
- If you do not follow the study rules
- If the study is stopped by the sponsor or IRB .

**What are my rights in this study?**

Your participation in this study is voluntary. No matter what decision you make, and even if your decision changes, there will be no penalty to you. You will not lose medical care or any legal rights. If you have any questions, concerns, or complaints regarding this study now or in the future, contact the principal investigator listed at the beginning of the form.

This research is being overseen by an Institutional Review Board (“IRB”). You may talk to the IRB at (503) 494-7887 or [irb@ohsu.edu](mailto:irb@ohsu.edu) if:

- Your questions, concerns, or complaints are not being answered by the research team
- You want to talk to someone besides the research team
- You have questions about your rights as a research subject
- You want to get more information or provide input about this research.

You may also submit a report to the OHSU Integrity Hotline online at <https://secure.ethicspoint.com/domain/media/en/gui/18915/index.html> or by calling toll-free (877) 733-8313 (anonymous and available 24 hours a day, seven days a week).

You do not have to join this or any research study. You do not have to allow the use and disclosure of your health information in the study, but if you do not, you cannot be in the study.

If you do join the study and later change your mind, you have the right to quit at any time. This includes the right to withdraw your authorization to use and disclose your health information. If you choose not to join any or all parts of this study, or if you withdraw early from any or all parts of the study, there will be no penalty or loss of benefits to which you are otherwise entitled, including being able to receive health care services or insurance coverage for services. Talk to the investigator if you want to withdraw from the study. If your spouse/partner is unable or unwilling to continue exercise training, you will be allowed to continue in the exercise program.

If you no longer want your health information to be used and disclosed as described in this form, you must send a written request or email stating that you are revoking your authorization to:

Knight Cancer Institute Clinical Trials

Attn: CRQA Assistant Director

Mail Code: KR-CRQA

3181 SW Sam Jackson Park Road

Portland, OR 97239

Email: [trials@ohsu.edu](mailto:trials@ohsu.edu)

Your request will be effective as of the date we receive it. However, health information collected before your request is received may continue to be used and disclosed to the extent that we have already taken action based on your authorization.

Your health care provider may be one of the investigators of this research study and, as an investigator, is interested in both your clinical welfare and in the conduct of this study. Before entering this study or at any time during the research, you may ask for a second opinion about your care from another doctor who is in no way involved in this project. You do not have to be in any research study offered by your physician.

You will be told of any new information that might make you want to change your mind about continuing to be in the study.

**What are the costs of taking part in this study?**

There will be no cost to you or your insurance company to participate in this study.

You will not be paid for taking part in this study.

**What happens if I am injured or hurt because I took part in this study?**

If you believe you have been injured or harmed while participating in this research and require treatment, contact Dr. Kerri Winters-Stone at (503) 494-0813.

If you are injured or harmed by the study procedures, you will be treated. OHSU does not offer any financial compensation or payment for the cost of treatment if you are injured or harmed as a result of participating in this research. Therefore, any medical treatment you need may be billed to you or your insurance. However, you are not prevented from seeking to collect compensation for injury related to negligence on the part of those involved in the research. Oregon law (Oregon Tort Claims Act (ORS 30.260 through 30.300)) may limit the dollar amount that you may recover from OHSU or its caregivers and researchers for a claim relating to care or research at OHSU, and the time you have to bring a claim.

**What is commercial development and how does it affect me?**

Information about you or obtained from you in this research may be used for commercial purposes, such as making a discovery that could, in the future, be patented or licensed to a company, which could result in a possible financial benefit to that company, OHSU, and its researchers. There are no plans to pay you if this happens. You will not have any property rights or ownership or financial interest in or arising from products or data that may result from your participation in this study. Further, you will have no responsibility or liability for any use that may be made of your samples or information.

**Where can I get more information?**

You may visit the NCI Web site at <http://cancer.gov/> for more information about studies or general information about cancer. You may also call the NCI Cancer Information Service to get the same information at: 1-800-4-CANCER (1-800-422-6237).

A description of this clinical trial will be available on <http://www.clinicaltrials.gov/> as required by U.S. law. This website will not include information that can identify you. At most, the website will include a summary of the results. You can search this website at any time.

When visiting either of these websites, use the search term(s) “cancer survivor and exercise” to locate information on this trial.

If you want more information about this study, ask your investigator.

Who can answer my questions about this study?

You can talk to the investigator about any questions or concerns you have about this study or to report side effects or injuries. Outside of regular clinic hours, you can speak with an oncologist on-call. Refer to the beginning of this consent form for contact names and phone numbers.

# Signature
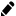


My Signature Agreeing to Take Part in the Study

| Your signature below indicates that you have read this entire form and that you agree to be in this study.  We will give you a copy of this signed form. | | |
| --- | --- | --- |
|  |  |  |
| **Participant Printed Name** | **Participant Signature** | **Date/Time** |
|  | | |
|  |  |  |
| **Person(s) Obtaining Consent Printed Name** | **Person(s) Obtaining Consent Signature** | **Date/Time** |
|  |  |  |

Use of an Interpreter: Complete if the participant is not fluent in English and an interpreter was used to obtain consent.  Participants who do not read or understand English must not sign this full consent form, but instead sign the short form translated into their native language.  This form should be signed by the investigator and interpreter only. If the interpreter is affiliated with the study team, the signature of an impartial witness is also required.

Print name of interpreter: ______________________________________

Signature of interpreter: ___________________________________  Date: _________

An oral translation of this document was administered to the participant in _____________ (state language) by an individual proficient in English and ____________ (state language).

If applicable:

Print name of impartial witness: __________________________________

Signature of impartial witness: ________________________________Date: _________

See the attached short form for documentation.
